# Polish Propolis—Chemical Composition and Biological Effects in Tongue Cancer Cells and Macrophages

**DOI:** 10.3390/molecules25102426

**Published:** 2020-05-22

**Authors:** Joanna Wezgowiec, Anna Wieczynska, Wlodzimierz Wieckiewicz, Julita Kulbacka, Jolanta Saczko, Natalia Pachura, Mieszko Wieckiewicz, Roman Gancarz, Kazimiera A. Wilk

**Affiliations:** 1 Department of Experimental Dentistry, Wroclaw Medical University, 50-425 Wroclaw, Poland; m.wieckiewicz@onet.pl; 2 Department of Engineering and Technology of Chemical Processes, Wroclaw University of Science and Technology, 50-370 Wroclaw, Poland; anna.wieczynska@pwr.edu.pl (A.W.); roman.gancarz@pwr.edu.pl (R.G.); kazimiera.wilk@pwr.edu.pl (K.A.W.); 3 Institute of Genetics and Microbiology, University of Wroclaw, 51-148 Wroclaw, Poland; 4 Department of Prosthetic Dentistry, Wroclaw Medical University, 50-425 Wroclaw, Poland; 5 Department of Molecular and Cellular Biology, Wroclaw Medical University, 50-556 Wroclaw, Poland; julita.kulbacka@umed.wroc.pl (J.K.); jolanta.saczko@umed.wroc.pl (J.S.); 6 Department of Chemistry, Wroclaw University of Environmental and Life Sciences, 50-375 Wroclaw, Poland; natalia.pachura@upwr.edu.pl

**Keywords:** total phenolic content, total flavonoid content, GC-MS, DPPH, antioxidant, anticancer agent, anti-inflammatory agent, gingival fibroblasts, oral cancer, natural extract

## Abstract

The purpose of this study was to compare the chemical composition and biological properties of Polish propolis. Ethanol, ethanol-hexane, hexane and hexane-ethanol extracts of propolis from three different regions of Poland were prepared. On the basis of the evaluation of their chemical composition as well as the extraction yield and free radical scavenging activity, the ethanol and hexane-ethanol extractions were proposed as the most effective methods. Subsequently, the biological properties of the extracts were evaluated to investigate the selectivity of an anticancer effect on tongue cancer cells in comparison to normal gingival fibroblasts. The obtained products demonstrated anticancer activity against tongue cancer cells. Additionally, when the lowest extract concentration (100 µg/mL) was applied, they were not cytotoxic to gingival fibroblasts. Finally, a possible anti-inflammatory potential of the prepared products was revealed, as reduced mitochondrial activity and proliferation of macrophages exposed to the extracts were observed. The results obtained indicate a potential of Polish propolis as a natural product with cancer-selective toxicity and anti-inflammatory effect. However, further studies are still needed to thoroughly explain the molecular mechanisms of its action and to obtain the promising health benefits of this versatile natural product.

## 1. Introduction

Nature, as an immemorial source of diverse active molecules, continues to serve as a major inspiration for drug development. Therapeutic applications of natural products offer great opportunities for modern medicine, while being simultaneously a huge challenge due to the problem of standardization procedures and the chemical complexity of these substances. On the other hand, such complexity is inevitable and a final therapeutic effect of a whole extract in general is better than effects of individual compounds since it results from the synergistic activity of the extract components [1].

One of the most attractive natural products is propolis—the resinous substance collected by bees from plants and mixed with wax and enzymes. It is then used to strengthen and protect their hives as well as to prevent decomposition of intruders’ carcasses. People have also widely used propolis in folk medicine, as it is known for a broad spectrum of biological properties including antibacterial, antifungal, antiviral, anti-inflammatory, antioxidant and anticancer activity [2]. Nowadays, it is used in the cosmetics industry, i.e., as a component of anti-acne creams and products for oral hygiene [3]. However, the therapeutic potential of propolis is still untapped and many research groups continue investigation of a chemical composition and biological properties of this material. The studies revealed a variability in propolis composition depending on the geographical region of collection and the plant sources. For instance, bud exudates of different poplar buds are the main source of propolis collected in the temperate zone, including Europe [4]. Silva-Carvalho et al. reported that poplar propolis is mainly composed of flavonoids, phenolic acids and its esters [3].

In particular, contemporary oral medicine may benefit from the wide spectrum of propolis activities. Many dental specialties which make use of this natural product have been reported [5]. Research on Polish propolis is mainly focused on its antimicrobial properties [6,7,8,9,10,11]. Interestingly, no research on the use of Polish propolis against oral cancer has been published so far. There is little research concerning the antiproliferative effect of Polish propolis on glioblastoma cells [12], colon, lung and breast cancer cells [13], as well as prostate cancer cells [14]. On the other hand, taking into account global data, the problem of oral cancer treatment is still unsolved. In 2018, new cases of oral cancer occurred globally in approximately 355,000 people and caused 177,000 deaths. The most common oral cancer type is tongue squamous cell carcinoma (TSCC), characterized by high lymphatic metastasis, recurrence and drug resistance. The current treatment approaches include surgery, which may be followed by radiotherapy and/or chemotherapy. However, there is still no effective therapeutic strategy and the death toll linked to this disease is still increasing [15].

The purpose of this study was to evaluate the anticancer properties of three different types of propolis from different regions of Poland on the in vitro model of tongue cancer cells. For this reason, ethanol, ethanol-hexane, hexane and hexane-ethanol extracts of Polish propolis were prepared. Normal human gingival fibroblasts were used as a control group of non-cancer cells and a murine macrophage-like cell line was used to evaluate anti-inflammatory potential of the prepared products. Additionally, chemical composition and antioxidant activity of the prepared extracts were compared.

## 2. Results

### 2.1. Extraction Yield

The extraction yields of the propolis extracts were calculated and are presented in Table 1. The extraction yield values of the ethanol extracts of propolis (EEP) were higher than the hexane extracts of propolis (HEP) and the highest values of extraction yield were obtained for propolis from Masovia (P2) and West Pomerania Province (P3). Therefore, the results indicated that ethanol was a better solvent than *n*-hexane. In addition, the hexane-ethanol extracts of propolis (HEEP) had the second highest extraction yields among all the propolis extracts analyzed.

### 2.2. Total Polyphenol Content

The total polyphenol content (TPC) was determined with the Folin–Ciocalteu method (Table 2). The statistical analysis revealed that there was not strong variation between the TPC of all propolis harvested in different provinces of Poland (*F*(2, 109) = 0.86794; *p* = 0.42270). However, regardless of a type of propolis, there were statistically significant differences of TPC (*F*(3, 108) = 1178.4; *p* = 0.0000) between different extracts, such as ethanol extract of propolis (EEP), ethanol-hexane extract of propolis (EHEP), hexane extract of propolis (HEP) and hexane-ethanol extract of propolis (HEEP). The TPC for EEP and HEEP was above 220 mg GAE (gallic acid equivalent)/g of the propolis extract, while the TPC for EHEP and HEP was below 50 mg GAE/g. Tukey’s post-hoc test revealed that all differences of TPC between each type of propolis extract tested were statistically significant at *p* < 0.05. Interestingly, when only EEP and HEEP were considered, the strong differences among TPC of the propolis harvested in different provinces were observed (*F*(2, 51) = 31.058; *p* = 0.00000). Thus, the propolis extracts from West Pomerania Province (P3) had the highest TPC, while the lowest TPC was obtained for propolis extracts from Podlasie (P1).

### 2.3. Total Flavonoid Content

The total flavonoid content (TFC), evaluated via aluminum chloride method, was presented in Table 2. Similarly to the measurement results of TPC, this analysis also revealed no statistically significant differences among propolis of different origin (*F*(2, 133) = 3.3270; *p* = 0.03891). On the other hand, differences among various extracts—EEP, EHEP, HEP and HEEP—were statistically significant (*F*(3, 132) = 360.77; *p* = 0.0000). Tukey’s post-hoc test revealed that all differences of TFC between each extract type tested were statistically significant at *p* < 0.05, except for the differences between EEP and HEEP samples (*p* = 0.122385). For all the ethanol and hexane-ethanol extracts (EEP and HEEP) analyzed, TFC was above 18.76 mg QE (quercetin equivalent)/g of the propolis extract, while ethanol-hexane and hexane extracts (EHEP and HEP) were characterized by significantly lower TFC. The highest TFC among all samples tested was found for propolis extracts from Masovia (EEP_P2 and HEEP_P2).

### 2.4. GC-MS Analysis

The chemical composition of EEP from different regions of Poland (Podlasie, Masovia and West Pomerania Province) was determined using gas chromatography–mass spectrometry (GC-MS) and is presented in Appendix A, Table A1. Briefly, the analysis of EEP revealed the presence of seventy-two components, out of which sixty-two were identified. The main components of the material analyses were TMS derivatives of 4-coumaric acid, d-fructose, d-glucose, d-mannopyranose, benzoic acid, lignoceric acid, ferulic acid and naringenin. GC-MS analysis of the ethanol extracts of propolis from Podlasie (EEP_P1) and Masovia (EEP_P2) showed a higher concentration of aromatic acids than the ethanol extract of propolis from West Pomerania Province (EEP_P3). The concentration of the compounds selected is presented in Table A1. The results indicate that the highest concentration of 4-coumaric acid and caffeic acid was measured in EEP_P2, while the lowest one was found in EEP_P3. Furthermore, the highest concentration of ferulic acid and benzoic acid was measured in EEP_P1, while the lowest one was found in EEP_P3.

The chemical composition of HEP from different regions of Poland is presented in Appendix A, Table A2. The profile of the compounds of the n-hexane extracts of propolis, determined by GC-MS, contains forty-one compounds (out of which forty were identified). The results showed domination of waxes and fatty acids derivatives of TMS. The main compounds of HEP_P1 and HEP_P2 were methyl triacontyl ether, heptacosane, pentacosane and lignoceric acid. The main compounds of HEP_P3 were heptacosane, lignoceric acid, 13-octadecanoic acid and methyl triacontyl ether. In addition, HEPs contained around two times more (HEP_P1: 8.10%, HEP_P2: 8.47%) or four times more (HEP_P3: 13.19%) benzoic acid than EEP.

The chemical composition of HEEP harvested from different regions of Poland is presented in Appendix A, Table A3. The profile of the compounds of the hexane-ethanol extracts of propolis contained sixty-five compounds (out of which sixty-two were identified). High similarity of the content of EEP and HEEP was observed. The dominant compounds in HEEP were 4-coumaric acid, d-fructose, d-glucose, d-mannopyranose and ferulic acid.

#### Fatty Acids Composition

The complete chemical composition of fatty acids in HEP from different regions of Poland is presented in Appendix A, Table A4. Fourteen compounds were identified when analyzing fatty acids contained in propolis of different origins. The main components found in the HEP fraction are: hexadecanoic acid methyl ester, heptadecanoic acid methyl ester, oleic acid methyl ester and tetracosanoic acid methyl ester.

### 2.5. DPPH Free Radical Scavenging Activity

**Figure 1:**
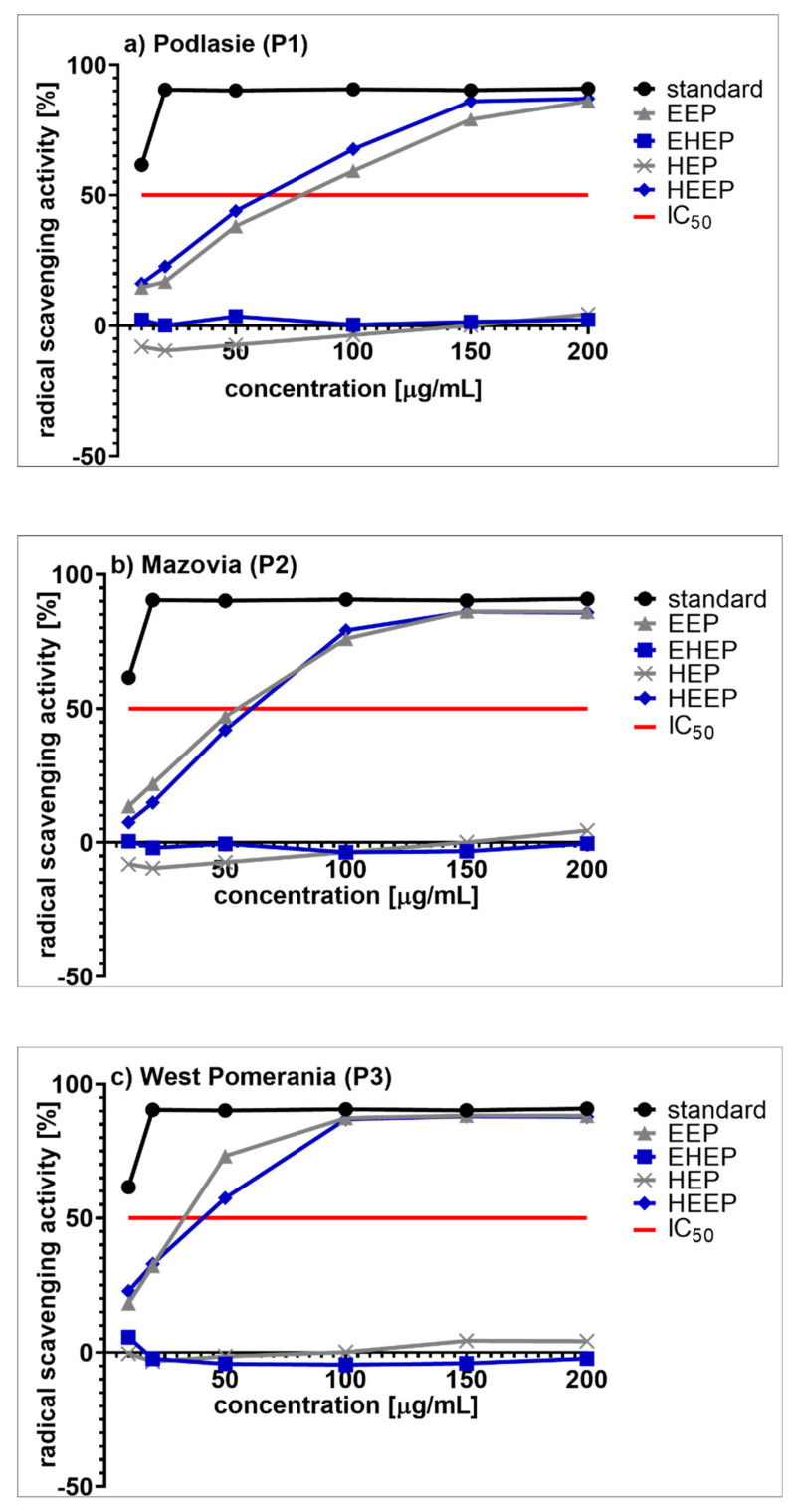
DPPH free radical scavenging activity of the prepared propolis extracts from: (**a**) Podlasie (P1); (**b**) Masovia (P2); (**c**) West Pomerania Province (P3); EEP— ethanol extract of propolis, EHEP—ethanol-hexane extracts of propolis, HEP—hexane extract of propolis, HEEP—hexane-ethanol extracts of propolis.

A 2,2-diphenyl-1-picrylhydrazyl (DPPH) assay was used to measure antioxidant activity of the propolis extracts, with the results presented in Figure 1.

As shown in the Figure 1a–c, all the tested ethanol-hexane and hexane extracts (EHEP and HEP) obtained from propolis harvested in different regions of Poland (P1, P2, P3) had only minimal DPPH free radical scavenging activity compared to the standard. Therefore, they were assumed as having no effect at all. In contrast, for EEP and HEEP, the free radical scavenging activity increased with the increase of the extracts’ concentration from 0 to 200 µg/mL. For these active extracts their IC_50_ were calculated (the concentration of extracts that inhibits the formation of DPPH free radicals by 50%) and showed in Table 3. Statistically significant differences among IC_50_ of propolis from different regions of Poland were demonstrated (*F*(2, 55) = 43.365; *p* = 0.00000). Tukey’s post-hoc test revealed that all differences of IC_50_ between each type of propolis tested (P1, P2, P3) were statistically significant at *p* < 0.05. Regardless of the type of propolis studied, the type of extract did not significantly influence the obtained values of IC_50_ (*F*(1, 56) = 0.09896; *p* = 0.75425). The lowest IC_50_ values were calculated for propolis extracts from West Pomerania Province (P3), indicating the highest antioxidant potential of these preparations among all the extracts tested.

### 2.6. Anticancer Activity

**Figure 2:**
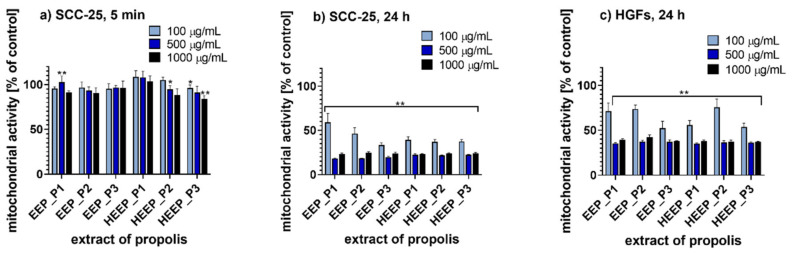
MTT assay results for: (**a**) tongue cancer cells (SCC-25) incubated for 5 min with propolis extracts; (**b**) tongue cancer cells (SCC-25) incubated for 24 h with propolis extracts; (**c**) human gingival fibroblasts (HGFs) incubated for 24 h with propolis extracts; * *p* < 0.05, ** *p* < 0.005; P1—propolis from Podlasie, P2—propolis from Masovia, P3—propolis from West Pomerania Province, EEP—ethanol extract of propolis, HEEP—hexane-ethanol extracts of propolis.

**Figure 3:**
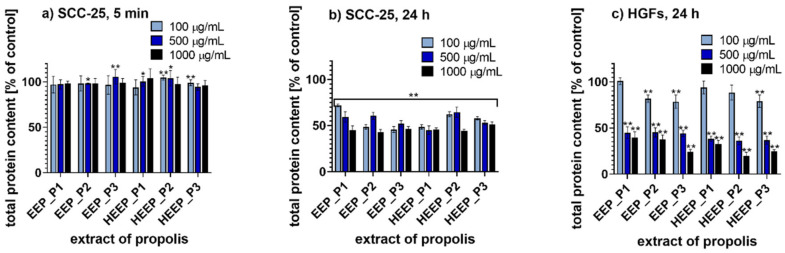
Sulforhodamine B (SRB) assay results for: (**a**) tongue cancer cells (SCC-25) incubated for 5 min with propolis extracts; (**b**) tongue cancer cells (SCC-25) incubated for 24 h with propolis extracts; (**c**) human gingival fibroblasts (HGFs) incubated for 24 h with propolis extracts; * *p* < 0.05, ** *p* < 0.005; P1—propolis from Podlasie, P2—propolis from Masovia, P3—propolis from West Pomerania Province, EEP—ethanol extract of propolis, HEEP—hexane-ethanol extracts of propolis.

The anticancer activity of the selected Polish propolis extracts was evaluated on human squamous cell carcinoma derived from tongue (SCC-25) after incubation for 5 min and 24 h. For this purpose, 3-(4,5-dimethyl-2-thiazolyl)-2,5-diphenyl-2*H*-tetrazolium bromide assay (MTT assay) and sulforhodamine B assay (SRB assay) were performed. In addition, for both methods, 24 h incubation with human gingival fibroblasts (HGFs) was used as a control model to investigate the effects of propolis in normal, i.e., non-cancer cells. The cytotoxicity values of EEP and HEEP harvested from three different regions in Poland and applied at three concentrations (100, 500 and 1000 µg/mL) are presented in Figure 2 (MTT assay results) and in Figure 3 (SRB assay results).

#### 2.6.1. MTT Assay

When 5 min of incubation with the propolis extracts was applied, mitochondrial activity of SCC-25 cells was only slightly reduced (Figure 2a). Moreover, when concentrations of all the extracts tested were increased, the mitochondrial activity was still above 80% compared to the control. However, the prolonged 24 h incubation period affected the cell viability significantly (Figure 2b). Three-way ANOVA revealed that for all tested extracts of Polish propolis the differences between groups based on the propolis type or extraction type were not statistically significant (*p* = 0.093920 and *p* = 0.493920, respectively). The only factor determining significant differences between groups was the extract concentration (*p* = 0.000000). For tongue cancer cells, incubation with each of the tested propolis extract at a concentration of 500 and 1000 µg/mL resulted in a decrease of mitochondrial activity to ca. 20% of the control. When the concentration of the propolis extracts applied was 100 µg/mL, the mitochondrial activity was most reduced for EEP_P3 (33% of the control) and least reduced for EEP_P1 (59% of the control), therefore, EEP_P1 was less active. The results obtained for HGFs treated with propolis indicated that the tested propolis extracts impaired also the viability of normal cells (Figure 2c). Incubation of HGFs with each tested propolis extract at a concentration of 500 and 1000 µg/mL reduced the mitochondrial activity to ca. 40% compared to the control. In addition, when the propolis extract concentration of 100 µg/mL was applied, EEP_P3 was the most active propolis extract, which reduced the mitochondrial activity to 52% of the control. Furthermore, HEEP_P2 reduced the mitochondrial activity to 76% compared to the control and therefore it was the least active propolis extract. Three-way ANOVA results for HGFs revealed that all the factors studied (type of propolis, type of extract and extract concentration) were source of significant variation at *p* < 0.05.

#### 2.6.2. SRB Assay

When SCC-25 cells were incubated with Polish propolis for 5 min, for all the concentrations of all the extracts tested the total protein content of cells was above 93% compared to the control (Figure 3a). Therefore, no cytotoxic effect of propolis extracts after a short-time incubation was revealed. However, the prolonged incubation, i.e., 24 h, affected the cellular proliferation significantly (Figure 3b). The results showed that 24 h incubation of tongue cancer cells with increasing concentration of propolis extract resulted in a decrease of total protein content. For example, when the concentrations of 100 and 500 µg/mL of all Polish propolis extracts were applied, cellular protein content was reduced to ca. 55% of the control. Notably, the least activity was observed at 100 µg/mL of EEP_P1, that reduced the cellular protein content to 72% compared to the control. However, for 500 µg/mL of HEEP_P1 the cellular protein content was reduced to 45% of the control. Finally, when tongue cancer cells were incubated with each of the Polish propolis extracts tested at a concentration of 1000 µg/mL, it resulted in a decrease in total protein content to ca. 45% compared to the control. The results for HGFs indicated that the analyzed propolis extracts at concentrations of 500 and 1000 µg/mL impaired the proliferation of normal cells (Figure 3c). Incubation of HGFs with 1000 µg/mL propolis extracts reduced total protein content to ca. 30% compared to the control. Incubation of HGFs with 1000 µg/mL of HEEP_P2 resulted in the lowest level of protein content, reduced to 20% of the control. On the other hand, incubation of HGFs with 1000 µg/mL of EEP_P1 resulted in the highest level of total protein content, i.e., 40% of the control. Additionally, incubation of HGFs with 500 µg/mL propolis extracts also induced a significant cytotoxicity. In contrast, for the concentration of 100 µg/mL, the lowest level of total protein content, i.e., 79% of the control, was observed when HGFs were incubated with EEP_P3 and HEEP_P3. The three-way ANOVA of results both for SCC-25 and HGFs revealed that all the factors studied (type of propolis, type of extract and extract concentration) were sources of significant variation at *p* < 0.05.

### 2.7. Anti-Inflammatory Potential

**Figure 4:**
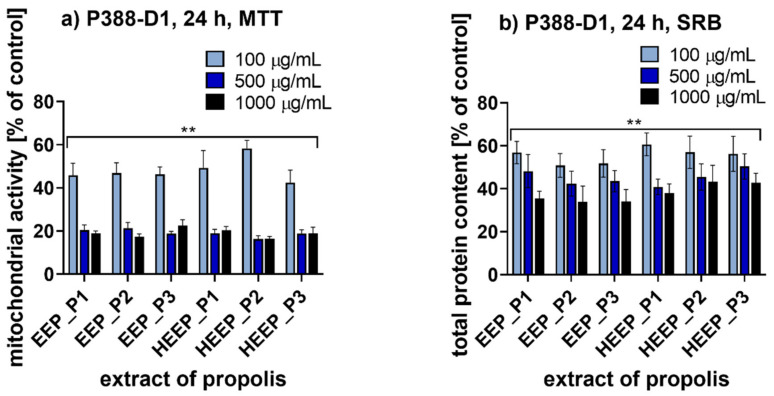
Results for murine macrophage cells (P388-D1) incubated for 24 h with propolis extracts; (**a**) MTT assay; (**b**) SRB assay; ** *p* < 0.005; P1—propolis from Podlasie, P2—propolis from Masovia, P3—propolis from West Pomerania Province, EEP—ethanol extract of propolis, HEEP—hexane-ethanol extracts of propolis.

Anti-inflammatory potential of the propolis extracts selected was evaluated on murine macrophage-like cell line (P388-D1) via MTT assay (Figure 4a) and SRB assay (Figure 4b) after 24 h of incubation. For all the analyzed concentrations of all the extracts tested it was observed that the prolonged 24 h incubation period affected the cellular mitochondrial activity and proliferation significantly. Incubation with each tested propolis extract at a concentration of 1000 µg/mL resulted in a decrease in mitochondrial activity of P388-D1 cells to ca. 19% of the control (Figure 4a) and a decrease in total protein content to ca. 38% of the control (Figure 4b). When the lowest concentration of extracts (100 µg/mL) was applied, the cellular mitochondrial activity was reduced to 48% and the cellular protein content to ca. 55% of the control.

## 3. Discussion

Propolis demonstrated antiproliferative activity on various cancer cell lines. It was reported that this natural product can block specific oncogene signaling pathways, leading to a decrease in cell proliferation. It can also increase apoptosis, exert antiangiogenic effects, and modulate the tumor microenvironment [3,16].

In spite of these beneficial properties, research on the anticancer activity of propolis on human tongue cancer cells is very limited. Antiproliferative activity of the ethanol extract of Chilean propolis on human mouth epidermoid carcinoma cells (KB) was demonstrated by Russo et al. [17]. Furthermore, Yen et al. and Chiu et al. showed an anti-inflammatory effect of various propolis extracts by inhibiting one of the inflammatory markers—COX-2—in KB cell line [18]. The study of Salehi et al. determined the chemopreventive effect of Iranian propolis on dysplastic changes in the rats’ tongue epithelium after administration of carcinogens (DMBA). The results have showed that propolis can prevent DMBA-induced dysplasia of the oral mucosa in animal model [19]. A similar effect was obtained for hydroalcoholic extract of Brazilian red propolis (HERP) on oral squamous cell carcinoma (OSCC) in rodents. The research revealed that HERP inhibited tumor growth and progression [20].

The anticancer effect of propolis is often attributed to one of its active components—caffeic acid phenethyl ester (CAPE). It can be considered as a potential support for therapy of patients with oral squamous cell carcinoma due to the ability to inhibit cellular proliferation and to prevent cancer metastasis [21,22,23]. On the other hand, the other approach to the clarification of the natural drug’s mechanisms of action is more comprehensive and takes into account a complexity of the product rather than the effect of its individual components. The study of Czyżewska et al. suggested that the synergistic effect of different polyphenols (chrysin, galangin, pinocembrin, caffeic acid, p-coumaric acid and ferulic acid) is responsible for the propolis’ ability to inhibit the growth of human tongue cancer cells through apoptosis [24]. Another study indicated the synergistic effect of the main components of Iranian propolis on mouth epidermoid carcinoma (KB) cells. MTT assay revealed that IC_50_ values of EEP and its main component, quercetin (Q) were 40 μg/mL and 195 μg/mL respectively after 48 h of incubation [25].

In this study, the whole extracts of Polish propolis were evaluated in terms of the selectivity of their anticancer effect on the tongue cancer cells in comparison to the normal gingival fibroblasts. Chemical analyses revealed that ethanolic and hexane-ethanol extraction were the most effective methods of raw propolis extraction to receive the most chemically complex product. This conclusion confirms the findings of the other studies indicating ethanol extraction as the most common method of raw propolis processing [26,27,28]. The second proposed method—ethanol–hexane extraction—may be an interesting alternative allowing the wax content removal [29]. Both spectroscopic and chromatographic methods enabled determination of a chemical character of the extracts obtained. The chemical compounds identified in the prepared propolis extracts are analogous to the results described by Sahinler and Kaftanogl [30] as well as by Anjum et al. [31], showing high concentration of the aromatic acids, hydrocarbons, alcohols, polyphenols and fatty acids. The presence of phenolic compounds in the propolis extracts is particularly promising when its anticancer activity is considered [32].

The biological analysis of the selected systems showed that the prolonged 24 h incubation of cells with propolis significantly affected the cell viability measured via MTT and SRB assays. Differences between groups, based on the propolis type or extraction type, were not statistically significant. This may confirm the hypothesis that differences in the chemical composition of the extracts obtained did not influence the general biological effect induced by them. It should be emphasized that higher concentrations of the propolis extracts (500 and 1000 µg/mL) significantly affected the viability of normal HGFs as well. For this reason, only the extract concentration of 100 µg/mL could be considered as effective selectively in cancer cells. Similar results demonstrating the cytotoxic effect of propolis on normal human fibroblasts were obtained by Tyszka-Czochara et al. [33], Popova et al. [13] and in our previous study [10]. Moreover, the study presented by Popova et al. revealed the similar chemical profile of the propolis sample (mainly flavanones and dihydroflavonols, as well as a series of esters of p-coumaric acid, ferulic acid, benzoic acid and fatty acids (palmitic acid, linoleic acid, oleic acid) compared to the extracts analyzed in our study [13]).

Additionally, due to the polyphenolic content of propolis, the anti-inflammatory activity of the extracts prepared was verified on macrophage models commonly used in case of natural compounds [34,35]. Szliszka et al. suggested that phenolic compounds may be responsible for a crucial contribution of Brazilian green propolis in the modulation of chemokine-mediated inflammation [34]. In our study, the impairment of the cellular proliferation and mitochondrial activity observed in macrophage-like cell line (P388-D1) suggested a possible anti-inflammatory activity of the prepared extracts. Here we have observed that the effect was dependent on the cytotoxic effect of propolis extracts applied.

In the future, the preliminary results reported in this research should be used to select the ethanol and hexane-ethanol extraction as the most effective methods of propolis extraction to obtain chemically complex and biologically active products. The prepared extracts should become a subject of an in-depth analysis aimed at the identification of the most active components and at the investigation of a precise molecular mechanism of their anticancer and anti-inflammatory action. In addition, the selected natural extracts could be combined with conventional chemotherapeutic regimens in order to propose safer and more effective treatment of cancer [36]. Finally, functional polymer microparticles for encapsulation of biologically active compounds could be designed and manufactured [37].

## 4. Materials and Methods

### 4.1. Material

The research materials were propolis samples originating from three different regions in Poland (Table 4). Raw propolis was collected from beehives manually. Before processing it was stored at room temperature under dark conditions.

### 4.2. Extraction

**Figure 5:**
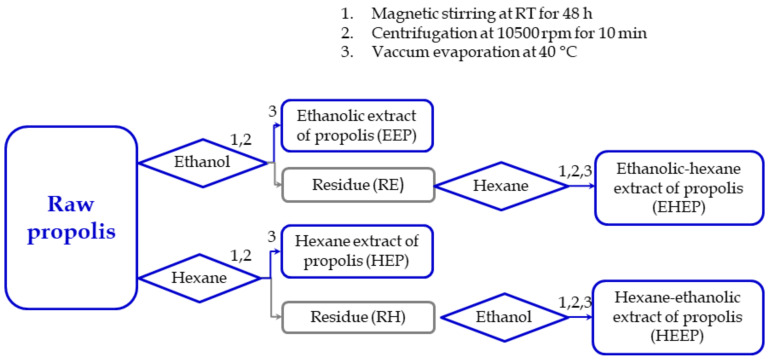
Schematic illustration of procedure for propolis extract preparation; EEP—ethanol extract of propolis, EHEP—ethanol-hexane extracts of propolis, HEP—hexane extract of propolis, HEEP— hexane-ethanol extracts of propolis, RE—residue after extraction with ethanol, RH—residue after extraction with hexane.

Ethanol, ethanol-hexane, hexane and hexane-ethanol extracts of Polish propolis were prepared according to the procedure illustrated in Figure 5. For this purpose, 5 g of raw propolis was cut into small pieces, dissolved in 50 mL of 70% ethanol (POCH, Poland) or 50 mL of hexane (POCH, Poland) and stirred for 48 h at room temperature under dark conditions, using a magnetic stirrer (Big-squid, IKA, Germany). Subsequently, the samples were centrifuged at 10,500 rpm for 10 min at room temperature, using a 5804 centrifuge (Eppendorf, Germany). The supernatant obtained was named ethanol extract of propolis (EEP) and hexane extract of propolis (HEP). Then, the residue was extracted one more time with ethanol or hexane to obtain EEP_II or HEP_II, respectively. Subsequently, the residue left after ethanol extraction was treated twice with hexane to obtain ethanol-hexane extracts (EHEP and EHEP_II). The residue left after hexane extraction was dissolved twice with 70% ethanol to obtain hexane-ethanol extracts (HEEP and HEEP_II). Non-dissolved residues were discarded.

The extracts were evaporated to dryness at 40 °C using a RV 10 rotary vacuum evaporator (IKA, Germany) and stored at 4 °C under dark conditions. After evaporation, the samples obtained were weighted using an analytical balance: WPS 510/C/2 (Radwag, Poland); extraction yields were expressed in percentage as a ratio of the mass of the sample after evaporation to the mass of the propolis material before extraction. The samples obtained after second extraction with the same solvent (EEP_II, HEP_II, EHEP_II, HEEP_II) were not subjected to further analysis due to their small quantity. Then, the samples were dissolved in methanol (POCH, Poland) at a concentration of 1 mg/mL (for chemical studies) or in DMSO (POCH, Poland) at a concentration of 100 mg/mL (for biological studies).

### 4.3. Total Polyphenol Content

The total soluble phenolic compounds in the samples were determined using the Folin–Ciocalteu colorimetric method [38]. For this purpose, 100 µL of analyzed propolis extract was dissolved in methanol (1 mg/mL) and then mixed with 900 µL of distilled water and 100 µL of Folin and Ciocalteu’s phenol reagent (Sigma-Aldrich, Poland). After 5 min of incubation, 1 mL of 7% Na_2_CO_3_ (POCH, Poland) and 400 µL of distilled water were added. Subsequently, the mixture was incubated for 2 h and the absorbance was measured at 765 nm using a UV-Vis spectrophotometer: SP 8001 (Metertech, Norway). Gallic acid (Sigma-Aldrich, Poland) was used as a standard. The results were expressed in mg of gallic acid equivalent per g of propolis extract (mg GAE/g). The minimum number of measurements for each extract was n = 9.

### 4.4. Total Flavonoid Content

The total flavonoid contents in the samples were determined using an aluminum chloride method [39]. Briefly, 100 µL of propolis extract dissolved in methanol (1 mg/mL) was mixed with 100 µL of 2% AlCl_3_ (Sigma-Aldrich, Poland). After 15 min of incubation, the absorbance was measured at 435 nm using a UV-Vis spectrophotometer: SP 8001 (Metertech, Norway). Quercetin (Sigma-Aldrich, Poland) was used as a standard. The results were expressed in mg of quercetin equivalent per g of propolis extract (mg QE/g). The minimum number of measurements for each extract was n = 9.

### 4.5. GC-MS Analysis

The propolis extracts obtained (EEP, HEP and HEEP) were evaluated in terms of a low-molecular-weight compound content by means of derivatization with N,O-bis (trimethylsilyl)trifluoroacetamide (BSTFA) silylation approach on gas chromatography, coupled with mass spectrometry (Shimadzu GC-MS QP 2020, Shimadzu, Kyoto, Japan). Each of the extracts was evaporated under reduced pressure. Then, 500 µL of pyridine and 50 µL of BSTFA were added to all samples. The mixture was placed in a vial and heated for 15 min at 70 °C. Separation was achieved using Zebron ZB-5 capillary column with a length of 30 m, inner diameter of 0.25 mm, and film thickness of 0.25 μm (Phenomenex, Torrance, CA, USA). The GC-MS analysis was performed according to the following parameters: scan mode with mass range from 40 to 1050 *m*/*z* in electronic impact (EI) mode at 70 eV; mode at 10 scan s^−1^ mode. Analyses were conducted using helium as a carrier gas at a flow rate of 1.0 mL min^−1^ in a split ratio of 1:20 and the following program: (a) 100 °C for 1 min; (b) rate of 2.0 °C min^−1^ from 100 to 190 °C; (c) rate of 5 °C min^−1^ from 190 to 300 °C. An injector was held at 280 °C, respectively. Compounds were identified by using two different analytical methods that compare: retention times with authentic chemicals (Supelco C7-C40 Saturated Alkanes Standard), and obtained mass spectra with available library data (Willey NIST 17, match index >90%).

#### Fatty Acids Composition

The lipid fraction was obtained according to the previously described method [40]. In the next step, the extracted nonpolar fraction, approx. 30 mg, was saponified (10 min at 75 °C) with 2 mL of 0.5 M KOH/MeOH solution and subjected to methylation (10 min at 75 °C) using 2 mL of 14% (*v*/*v*) BF3/MeOH (Sigma-Aldrich, St. Louis, MO, USA). Subsequently, water was added to reaction mixture and methyl esters of fatty acids were extracted with 10 mL of hexane (UQF Wroclaw, Poland), then washed with 10 mL 10% sodium bicarbonate (UQF Wroclaw, Poland) and desiccated with anhydrous sodium sulphate. The organic phase was evaporated under reduced pressure and stored at −27 °C until chromatographical analysis. The FAME profile was assessed using gas chromatograph coupled with a mass spectrometer (Shimadzu GCMS QP 2020, Shimadzu, Kyoto, Japan). Separation was achieved using Zebron ZB-FAME capillary column with a length of 60 m, inner diameter of 0.20 mm, and film thickness of 0.20 μm (Phenomenex, Torrance, CA, USA). The GC-MS analysis was according to the following parameters: scan mode with mass range from 40 to 400 *m*/*z* in electronic impact (EI) mode at 70 eV; mode at 3 scan s^−1^ mode. Analyses were conducted using helium as a carrier gas at a flow rate of 1.8 mL min^−1^ in a split ratio of 1:10 and the following program: (a) 80 °C for 2 min; (b) rate of 3.0 °C min^−1^ from 80 to 180 °C; (c) rate of 8 °C min^−1^ from 180 to 240 °C. An injector was held at 280 °C, respectively. Compounds were identified by using two different analytical methods that compare: retention times with authentic chemicals (Supelco 37 Component FAME Mix), and obtained mass spectra with available library data (Willey NIST 17, match index >90%).

### 4.6. DPPH Free Radical Scavenging Activity

The 2,2-diphenyl-1-picrylhydrazyl (DPPH) free radical scavenging activity was determined using a method described by Yang et al. [39]. For this purpose, 100 µL of propolis extract dissolved in methanol (10, 20, 50, 100, 150, 200 µg/mL) was placed into a 96 well plate (Nunc, Denmark) and 100 µL of 0.2 mM DPPH solution (Sigma-Aldrich, Poland) was added. After 15 min of incubation, the absorbance was measured at 517 nm using a multiwell-plate reader (EnSpire Multimode Reader, Perkin Elmer, USA). Ascorbic acid (P.P.H. STANLAB Sp.J., Poland) was used as a standard. The percentage inhibition capacity was calculated from the following equation:

percentage inhibition = (A_0_ − A_1_)/(A_0_ × 100),

where A_0_ is the absorbance of the control group and A_1_ is the absorbance of the extracts.

### 4.7. Biological Characterisation

Taking into account the results of the chemical analyses, hexane extracts (HEP) and ethanol-hexane extracts (EHEP) were excluded from further studies. Biological analyses were conducted only for ethanol extracts (EEP) and hexane-ethanol extracts (HEEP), which were characterized by the highest TPC, TFC and DPPH free radical scavenging activity.

#### 4.7.1. Cell Culture

Human squamous cell carcinomas derived from tongue (SCC-25 cell line, ATCC CRL-1628, ATCC, USA) were cultured in a 1:1 mixture of Dulbecco’s Modified Eagle’s Medium (DMEM) and Ham’s F12 medium (Lonza, Switzerland) supplemented with 10% fetal bovine serum (FBS, Sigma-Aldrich, Poland), and antibiotics: penicillin/streptomycin (Sigma-Aldrich, Poland), as recommended by ATCC.

Human gingival fibroblasts (HGFs) were mechanically isolated from a fragment of gingival tissue (1–2 mm) in healthy patients, according to the procedure described by Dominiak and Saczko [41]. The biopsies were provided by the Department of Dental Surgery at the Wroclaw Medical University in accordance with the requirements of the Bioethics Commission of Wroclaw Medical University (Bioethical Committee approval, No.: KB-8/2010). The fragment of tissue was taken by a scalpel and immediately placed on Petri dishes (60 mm, Nunc, Denmark) with DMEM (Sigma-Aldrich, Poland) containing 10% FBS (Sigma-Aldrich, Poland) and antibiotics: penicillin/streptomycin (Sigma-Aldrich, Poland).

Murine macrophage-like cells (P388-D1 cell line, ATCC CCL-46, ATCC, USA) were cultured in a 1:1 mixture of DMEM and RPMI 1640 medium (Lonza, Switzerland) supplemented with 10% FBS (Sigma-Aldrich, Poland) and antibiotics: penicillin/streptomycin (Sigma-Aldrich, Poland).

All cell lines were incubated in a humidified atmosphere at 37 °C and 5% CO_2_. After trypsinization with 0.25% trypsin-EDTA (Sigma-Aldrich, Poland), the cells were passaged and grown in 25 cm^2^ flasks (Equimed, Poland). In order to evaluate cytotoxicity of the extracts tested, cells were seeded into a 96-well plate (Nunc, Denmark). After 24 h, the culture medium was removed and then propolis extracts, diluted with an appropriate culture medium (100, 500 and 1000 μg/mL), were added for 5 min or 24 h. MTT and SRB assays were performed 24 h later. All results were referred to the untreated control cells.

#### 4.7.2. MTT Assay

To evaluate cytotoxicity of propolis extracts (EEP and HEEP) on the basis of differences in mitochondrial function, 3-(4,5-dimethyl-2-thiazolyl)-2,5-diphenyl-2*H*-tetrazolium bromide (MTT) assay was performed. Cells were incubated for 90 min with 100 μL of the MTT reagent (Sigma-Aldrich, Poland) at 37 °C. Then, formazan crystals were dissolved by addition of 100 μL of acidic isopropanol and by mixing. The absorbance was measured at 570 nm using a multiwell plate reader (EnSpire Multimode Reader, Perkin Elmer, USA). The results were expressed as the percentage of treated cells with altered mitochondrial function in relation to untreated control cells with normal mitochondrial activity, considered as 100%.

#### 4.7.3. SRB Assay

To evaluate cytotoxicity of propolis extracts on the basis of differences in total protein content in cells, sulforhodamine B (SRB) assay was performed. The protocol was based on the procedure described in [42]. Cell monolayers were fixed with 10% (vol/vol) trichloroacetic acid (Roth, Poland) for 1 h at 4 °C, subsequently washed (five times) in cold water and desiccated. Cell staining was performed for 30 min using 0.4% SRB (Sigma-Aldrich, Poland) in 1% acetic acid (Sigma-Aldrich, Poland) at room temperature. After incubation, the excess of dye was removed by means of washing with 1% (*v*/*v*) acetic acid (four times). Plates were desiccated and the protein-bound dye was dissolved in 10 mM Tris base solution (pH 10.5) (BioShop, Canada). The absorbance was measured at 490 nm using a multiwell plate reader (GloMax Discover, Promega, USA). The results were expressed as the percentage of total protein content in treated cells in relation to untreated control cells.

### 4.8. Statistical Analysis

The results are presented as means ± standard deviation (SD) values for minimum n = 9 repeats. The results were analyzed by one-way ANOVA and α = 0.05 using Statistica ver. 13.3 software (StatSoft, Poland). *F*-values and *p*-values were determined, the values *p* ≤ 0.05 were considered as statistically significant. Tukey’s HSD test was performed when ANOVA indicated statistically significant results. Additionally, for the MTT and SRB assays, the statistical significance of the differences between mean values of different groups and the untreated control group was evaluated by Student’s *t*-test. The values *p* ≤ 0.05 were marked with an asterisk and considered as statistically significant. Finally, for MTT and SRB assay results, three–way ANOVA test was performed to indicate, which factor (type of propolis, type of extract, extract concentration) determines significant differences between groups, *p* ≤ 0.05 were considered as statistically significant.

## 5. Conclusions

This study has revealed differences in chemical composition and antioxidant activity of the extracts of three different types of Polish propolis obtained after extraction with ethanol, hexane and combinations of both. The products selected (EEP and HEEP) demonstrated anticancer activity in the tongue cancer cells and cytotoxicity towards murine macrophages. In addition, EEP and HEEP did not have any cytotoxic effect in the normal gingival fibroblasts when the lowest concentration was applied.

The following conclusions can be drawn on the basis of the results obtained:

- The highest total extraction yields were obtained for ethanol and hexane-ethanol extracts (EEP and HEEP);
- Total polyphenol content (TPC) and total flavonoid content (TFC) of ethanol and hexane-ethanol extracts (EEP and HEEP) were much higher than TPC and TFC of ethanol-hexane and hexane extracts (EHEP and HEP);
- Antioxidant potential of ethanol and hexane-ethanol extracts (EEP and HEEP) was much higher than that of ethanol-hexane and hexane extracts (EHEP and HEP);
- The extracts selected (EEP and HEEP) demonstrated anticancer activity in the tongue cancer cells; 24 h incubation affected cell viability and cellular proliferation significantly;
- The propolis extracts tested at higher concentrations (500 and 1000 µg/mL) impaired the proliferation of normal cells as well;
- The observed cytotoxicity of the extracts prepared towards murine macrophages requires further investigation to evaluate their possible anti-inflammatory potential.

As a final conclusion, we can select the minimal dose of 100 µg/mL of the extracts applied, which caused anticancer effect on human tongue cancer cells with limited cytotoxic effect on normal mucosal cells and simultaneous anti-inflammatory potential. However, further studies on Polish propolis are still necessary in order to thoroughly explain the molecular mechanisms of its action and to obtain promising health benefits of this versatile natural product.

## Figures and Tables

**Table 1:** Extraction yields of the prepared extracts; P1—propolis from Podlasie, P2—propolis from Masovia, P3—propolis from West Pomerania Province, EEP—ethanol extract of propolis, EHEP— ethanol-hexane extracts of propolis, HEP—hexane extract of propolis, HEEP—hexane-ethanol extracts of propolis.

Symbol	Sequence of Solvents	Extraction Yield [%]		
		P1	P2	P3
**EEP**	ethanol	33.4	57.5	63.7
**EHEP**	ethanol–hexane	24.2	8.4	13.3
**HEP**	hexane	28.2	17.5	14.5
**HEEP**	hexane–ethanol	32.9	42.7	47.9

**Table 2:** Total polyphenol content and total flavonoid content of the prepared extracts; the results are expressed as mean ± SD; P1—propolis from Podlasie, P2—propolis from Masovia, P3—propolis from West Pomerania Province, EEP—ethanol extract of propolis, EHEP—ethanol-hexane extracts of propolis, HEP—hexane extract of propolis, HEEP—hexane-ethanol extracts of propolis, GAE—gallic acid equivalent, QE—quercetin equivalent.

Propolis Extract	P1	P2	P3
	**Total Polyphenol Content [mg GAE/g]**		
**EEP**	222.05 ± 14.29	259.63 ± 11.73	275.79 ± 13.42
**EHEP**	16.36 ± 1.12	19.60 ± 1.07	18.02 ± 1.09
**HEP**	20.45 ± 4.08	45.02 ± 7.22	38.84 ± 6.40
**HEEP**	249.92 ± 8.64	277.19 ± 14.28	308.92 ± 15.85
	**Total Flavonoid Content [mg QE/g]**		
**EEP**	18.76 ± 0.66	22.19 ± 0.44	19.79 ± 0.19
**EHEP**	11.10 ± 0.06	10.87 ± 0.03	12.99 ± 0.07
**HEP**	12.23 ± 0.21	13.49 ± 0.13	14.45 ± 0.19
**HEEP**	19.00 ± 0.57	22.46 ± 0.40	21.63 ± 0.25

**Table 3:** The concentration of extracts that inhibits the formation of DPPH free radicals by 50% (IC_50_); P1—propolis from Podlasie, P2—propolis from Masovia, P3—propolis from West Pomerania Province, EEP—ethanol extract of propolis, HEEP—hexane-ethanol extracts of propolis.

Propolis Extract	IC_50_ [µg/mL]		
	P1	P2	P3
**EEP**	78.02 ± 4.86	55.07 ± 7.39	33.01 ± 2.73
**HEEP**	62.84 ± 14.59	60.72 ± 2.89	40.92 ± 7.55

**Table 4:** Geographical origin of the Polish propolis examined.

Symbol	Region of Origin	The Most Abundant Plants in the Region	Bee Species
P1	Podlasie (Hajnowka)	spruce (*Picea abies L.*)—30%, pine (*Pinus sylvestris L.*)—27%, alder (*Alnus glutinosa L.*)—20%, sessile oak (*Quercus petraea L*.)—10%, silver birch (*Betula pendula L*.)—7%	*Apis mellifera carnica x Apis mellifera caucasica*
P2	Mazovia (Ciechanow)	pine (*Pinus sylvestris L*.)—70%, alder (*Alnus glutinosa L*.)—10%, sessile oak (*Quercus petraea L*.)—10%, silver birch (*Betula pendula L*.)—7%	*Apis mellifera carnica*
P3	West Pomerania (Miedzyzdroje)	pine (*Pinus sylvestris L*.)—75%, alder (*Alnus glutinosa L*.)—5%, beech (*Fagus sylvatica L*.)—5%, sessile oak (*Quercus petraea L*.)—5%, silver birch (*Betula pendula L*.)—4%	*Apis mellifera mellifera*

## Appendix A

**Table A1:** GC-MS profile of ethanol extracts of propolis (EEP) from different regions of Poland: *EEP_P1*—Podlasie (Hajnowka), *EEP_P2*—Masovia (Ciechanow), *EEP_P3*—West Pomerania Province (Miedzyzdroje).

	Substances	RT	RI _exp_	RI _lit_	EEP_P1[%]	EEP_P2[%]	EEP_P3[%]
1	Benzyl alcohol, TMS derivative	6.110	1155	1152	0.12	0.15	0.1
2	Benzoic Acid, TMS derivative	8.795	1246	1249	4.35	3.8	3.15
3	Glycerol, TMS	10.162	1287	1289	0.78	0.99	1.55
4	Butanedioic acid, 2TMS derivative	11.445	1320	1321	0.12	0.04	0.13
5	1-Monoacetin, 2O-TMS	11.740	1326	1324	0.13	0.07	0.08
6	4-Hydroxybenzaldehyde, TMS derivative	13.737	1373	1383	0.22	0.16	0.08
7	Hydroquinone, 2TMS derivative	15.308	1409	1408	0.24	0.2	0.08
8	Malic acid, 3TMS derivative	20.278	1511	1497	0.09	0.06	1.1
9	5-Oxoproline, TMS derivative	20.680	1521	1527	0.1	0.09	-
10	Vanillin, TMS derivative	21.622	1536	1530	1.48	0.5	0.37
11	Cinnamic acid, TMS derivative	22.048	1545	1542	0.12	0.14	0.25
12	4-Hydroxybenzoic acid, 2TMS derivative	26.623	1634	1635	0.27	0.22	0.08
13	Dodecanoic acid, TMS	27.858	1658	1655	0.13	0.09	-
14	β-D-Xylopyranose, 4TMS derivative	34.330	1784	1777	0.17	-	-
15	o-Coumaric acid, 2TMS derivative	34.975	1797	1815	0.26	0.26	0.09
16	D-Psicofuranose	37.465	1848	1837	1.28	1.2	0.76
17	D-Fructose, 5TMS derivative	37.855	1856	1867	7.4	9.55	8.51
18	D-Sorbitol, 6TMS derivative	41.075	1922	1920	0.22	0.09	7.15
19	D-Glucose, 5TMS derivative	41.635	1934	1928	4.82	8.5	-
20	4-Coumaric acid, 2TMS derivative	42.223	1947	1949	10.74	13.68	1.34
21	D-Glucitol, 6TMS derivative	43.902	1982	1980	0.44	0.34	0.18
22	Gallic acid, 4TMS derivative	44.118	1986	1987	0.39	0.1	0.21
23	Salicylic acid, trimethylsilyl ether, benzyl ester	45.910	2028	2025	0.55	0.49	0.11
24	D-Mannopyranose, 5TMS derivative	46.347	2038	2037	4.87	8.84	7.8
25	D-Gluconic acid, 6TMS derivative	46.875	2052	2043	0.16	0.16	0.09
26	Palmitic Acid, TMS derivative	47.070	2057	2050	0.85	0.7	0.69
27	Isoferulic acid, 2TMS derivative	48.435	2090	2081	0.17	0.67	2.7
28	Ferulic acid, 2TMS derivative	48.950	2103	2103	4.79	2.95	2.69
29	Myo-Inositol, 6TMS derivative	49.822	2132	2129	0.18	0.1	1.17
30	Phtalic acid derivative*	50.387	2150	-	1.07	1.19	4.02
31	Caffeic acid, 3TMS derivative	50.548	2157	2155	1.06	2.15	-
32	Unknown	51.908	2202	-	1.54	2.48	0.19
33	13-Octadecenoic acid, (E)-, TMS derivative	52.383	2222	2228	0.59	0.72	0.88
34	3,7,11,15-Tetramethyl-2,6,10,14-hexadecatetraene-1-ol trimethylsilyl ether	52.697	2236	2234	1.65	2.7	0.25
35	Tricosane	54.272	2300	2300	1.14	0.42	0.08
36	Unknown	55.733	2370	-	-	-	1.21
37	Unknown	56.810	2424	-	-	-	2.4
38	Pterostilbene, trimethylsilyl ether	57.507	2462	-	0.39	0.48	0.82
39	Pentacosane	58.223	2501	2506	0.83	0.26	0.64
40	Unknown	58.548	2519	-	4.41	4.32	1.5
41	Ethyl trans-caffeate, bis(tert-butyldimethylsilyl) ether	58.675	2527	2547	0.11	0.08	0.16
42	Bisphenol C*	59.085	2551	-	1.47	3.58	5.2
43	1-Docosanol, TMS derivative	59.220	2558	2557	0.25	0.48	0.67
44	Unknown	59.577	2579	-	0.3	0.19	0.45
45	Butanoic acid, 4-methoxy-2-nitro-, 2,6-bis(1,1-dimethylethyl)-4-methoxyphenyl ester	59.990	2604	2595	0.6	0.25	3.25
46	Unknown	60.465	2635	-	0.45	0.71	0.56
47	Behenic acid, TMS derivative	60.650	2645	2644	1.05	0.37	-
48	Unknown	60.938	2664	-	0.82	1.22	2.59
49	Unknown	61.132	2675	-	0.27	0.59	1.51
50	Unknown	61.280	2685	-	2.12	0.96	0.9
51	Maltose, 8TMS derivative, isomer 2	61.415	2693	2693	1.56	2.96	5.33
52	n-Heptacosane	61.527	2700	2700	0.82	0.2	-
53	Sucrose, 8TMS derivative	61.700	2712	2712	2.38	0.43	0.23
54	D-(+)-Turanose, octakis(trimethylsilyl) ether, methyloxime (isomer 1)	61.925	2727	2724	0.53	1.96	4.92
55	Maltose, OTMS	62.035	2735	2733	0.38	0.18	0.11
56	D-Cellobiose, (isomer 2), 8TMS derivative	62.258	2749	2762	1.7	2.64	0.43
57	Naringenin, O,O’-bis(trimethylsilyl)-	62.612	2772	2778	1.07	3.48	6.06
58	Unknown	63.180	2813	-	-	-	2.08
59	Isosakuranetin, TMS derivative	63.345	2821	2818	0.74	1.43	0.2
60	Lignoceric acid, TMS derivative	63.648	2842	2838	7.82	3.45	0.35
61	Sakuranetin, TMS derivative	64.200	2882	2877	0.96	0.58	0.26
62	Catechine, 5TMS derivative	64.872	2932	2938	0.17	0.13	-
63	Gettibiose, TMS derivative	65.712	2991	2989	0.39	0.38	0.29
64	Triacontane	65.933	3009	3003	0.44	0.36	0.27
65	Pectolinaringenin, TMS derivative	66.108	3021	3037	0.54	0.41	0.38
66	Hexacosanoic acid, TMS derivative	66.367	3041	3039	0.7	0.18	1.84
67	Nonacosan-10-ol, O-TMS	66.840	3078	3048	2.59	0.64	-
68	Nonacosan-9-ol, O-TMS	66.925	3085	3053	2.13	1.06	0.91
69	Hentriacontane	67.132	3100	3103	0.36	0.2	0.83
70	Kaempferol, 4TMS	67.298	3114	3112	0.29	0.86	0.77
71	Trimethylsilyl octacosanoate, TMS derivative	69.220	3256	3229	0.52	0.2	0.52
72	Methyl triacontyl ether	69.512	3275	3233	8.38	0.97	0.44

*RI* (retention time); *RI_exp_.* and *RI_lit_*. indicate retention indices based on experiments and literature, respectively.

**Table A2:** GC-MS profile of hexane extracts of propolis (HEP) from different regions of Poland: *HEP_P1*—Podlasie (Hajnowka), *HEP_P2*—Masovia (Ciechanow), *HEP_P3*—West Pomerania Province (Miedzyzdroje).

	Substances	RT	RI _exp_	RI _lit_	HEP_P1[%]	HEP_P2[%]	HEP_P3[%]
1	Benzoic Acid, TMS derivative	8.765	1246	1249	8.1	8.47	13.19
2	Glycerol, TMS	10.137	1287	1289	0.82	0.47	0.6
3	Decanoic acid, TMS derivative	17.830	1460	1450	0.15	1.5	0.14
4	Vanillin, TMS derivative	21.590	1536	1530	0.79	0.36	0.27
5	Cinnamic acid, TMS derivative	21.998	1545	1542	0.17	0.26	0.87
6	Dodecanoic acid, TMS	27.828	1658	1655	0.31	0.37	0.36
7	β-D-Xylopyranose, 4TMS derivative	34.300	1784	1777	0.25	1.36	-
8	D-Fructose, 5TMS derivative	37.770	1856	1867	0.36	0.36	0.38
9	4,7,10-Hexadecatrienoic acid, methyl ester	40.008	1899	1902	0.36	0.13	-
10	D-Sorbitol, 6TMS derivative	41.032	1922	1920	0.2	-	-
11	4-Coumaric acid, 2TMS derivative	42.122	1947	1949	0.33	0.19	0.21
12	Salicylic acid, trimethylsilyl ether, benzyl ester	45.865	2028	2025	0.87	1.84	0.63
13	Palmitic Acid, TMS derivative	47.035	2057	2050	2.48	3.06	2.96
14	Ferulic acid, 2TMS derivative	48.887	2103	2103	1.2	0.76	3.01
15	Phtalic acid derivative*	50.357	2150		1.04	1.31	0.21
16	Methyl caffeate, 2TMS derivative	51.882	2201	1997	1.02	1.67	0.07
17	13-Octadecenoic acid, (E)-, TMS derivative	52.365	2222	2228	1.7	4.3	8.05
18	3,7,11,15-Tetramethyl-2,6,10,14-hexadecatetraene-1-ol trimethylsilyl ether	52.677	2236	2234	0.77	1.21	-
19	Stearic acid, TMS derivative	53.047	2249	2246	0.55	0.89	1.15
20	Tricosane	54.255	2300	2300	3.55	2.67	3.05
21	Arachidic acid, TMS derivative	57.212	2446	2447	0.43	0.4	0.63
22	Pterostilbene, trimethylsilyl ether	57.723	2462	-	0.34	0.28	0.13
23	Pentacosane	58.200	2501	2506	5.24	4.6	4.00
24	Ethyl trans-caffeate, bis(tert-butyldimethylsilyl) ether	58.518	2527	2547	0.84	1.54	1.2
25	Bisphenol C*	59.043	2551		0.3	0.52	1.28
26	Behenic acid, TMS derivative	60.617	2643	2644	2.11	1.42	2.19
27	Unknown	61.255	2683	-	2.02	1.56	1.77
28	n-Heptacosane	61.512	2700	2700	14.11	14.31	12.45
29	Maltose, OTMS	62.007	2735	2733	0.38	0.19	0.1
30	D-Cellobiose, (isomer 2), 8TMS derivative	62.290	2749	2762	0.37	1.16	0.55
31	Octacosane	63.003	2798	2800	0.56	1.09	0.68
32	Lignoceric acid, TMS derivative	63.620	2842	2838	7.72	5.88	12.02
33	Sakuranetin, TMS derivative	64.170	2882	2877	0.53	-	0.23
34	Nonacosane	64.438	2899	2900	7.15	9.24	7.34
35	Hexacosanoic acid, TMS derivative	66.345	3041	3039	0.63	0.67	2.21
36	Nonacosan-10-ol, O-TMS	66.817	3078	3048	5.01	3.31	3.27
37	Nonacosan-9-ol, O-TMS	66.907	3085	3053	4.36	3.24	2.7
38	Hentriacontane	67.115	3100	3103	4.25	6.66	4.62
39	Myristic acid, 9-hexadecenyl ester, (Z)-	68.128	3177	3130	0.6	0.34	0.17
40	Trimethylsilyl octacosanoate, TMS derivative	69.188	3256	3229	1.21	1.01	0.5
41	Methyl triacontyl ether	69.493	3276	3233	16.8	11.36	6.82

*RI* (retention time); *RI_exp_.* and *RI_lit_.* indicate retention indices based on experiments and literature, respectively.

**Table A3:** GC-MS profile of hexane-ethanol extracts of propolis (HEEP) from different regions of Poland: *HEEP_P1*—Podlasie (Hajnowka), *HEEP_P2*—Masovia (Ciechanow), *HEEP_P3*—West Pomerania Province (Miedzyzdroje).

	Substances	RT	RI _exp_	RI _lit_	HEEP_P1[%]	HEEP_P2[%]	HEEP_P3[%]
1	Benzyl alcohol, TMS derivative	6.112	1155	1152	0.09	0.04	0.08
2	Benzoic Acid, TMS derivative	8.797	1246	1249	3.16	1.24	1.27
3	Cinnamaldehyde	9.905	1272	1274	0.04	0.08	0.06
4	Glycerol, TMS	10.170	1287	1289	0.87	1.05	1.8
5	Butanedioic acid, 2TMS derivative	11.465	1320	1321	0.12	0.05	0.18
6	1-Monoacetin, 2O-TMS	11.748	1326	1324	0.14	0.06	0.09
7	4-Hydroxybenzaldehyde, TMS derivative	13.748	1373	1383	0.26	0.19	0.14
8	Hydroquinone, 2TMS derivative	15.325	1409	1408	0.4	0.22	0.13
9	Cinnamyl alcohol, trimethylsilyl ether	16.273	1422	1428	-	0.02	0.03
10	Malic acid, 3TMS derivative	20.282	1511	1497	0.12	0.12	0.81
11	5-Oxoproline, TMS derivative	20.693	1521	1527	0.12	0.06	0.05
12	Vanillin, TMS derivative	21.640	1537	1530	1.82	0.46	0.27
13	Cinnamic acid, TMS derivative	22.043	1545	1542	0.07	0.05	0.15
14	3,4-Dihydroxybenzaldehyde,	26.045	1622	1612	-	0.13	0.27
15	4-Hydroxybenzoic acid, 2TMS derivative	26.638	1634	1635	0.48	0.22	0.18
16	Dodecanoic acid, TMS	27.668	1658	1655	0.14	0.02	0.08
17	β-D-Xylopyranose, 4TMS derivative	34.563	1784	1777	0.13	0.03	0.03
18	o-Coumaric acid, 2TMS derivative	34.968	1797	1815	0.28	0.25	0.09
19	4-Methoxycinnamic acid, TMS derivative	36.602	1830	1833	-	0.08	0.26
20	D-Psicofuranose	37.475	1848	1837	1.46	1.59	2.24
21	D-Fructose, 5TMS derivative	37.865	1856	1866	12.58	11.92	2.03
22	D-Sorbitol, 6TMS derivative	41.092	1922	1920	0.16	0.1	-
23	D-Glucose, 5TMS derivative	41.658	1934	1928	7.14	11.9	8.27
24	4-Coumaric acid, 2TMS derivative	42.252	1947	1949	16.74	13.52	10.74
25	D-Glucitol, 6TMS derivative	43.917	1982	1980	0.43	0.25	0.44
26	Gallic acid, 4TMS derivative	44.125	1987	1987	0.68	0.13	-
27	Salicylic acid, trimethylsilyl ether, benzyl ester	45.932	2028	2025	0.1	0.08	0.2
28	D-Mannopyranose, 5TMS derivative	46.353	2038	2037	6.33	13.13	9.13
29	D-Gluconic acid, 6TMS derivative	46.885	2052	2043	0.15	0.2	0.08
30	Palmitic Acid, TMS derivative	47.083	2057	2050	0.49	0.18	0.09
31	Isoferulic acid, 2TMS derivative	48.453	2090	2081	0.09	0.78	2.95
32	Ferulic acid, 2TMS derivative	48.965	2103	2103	7.93	3.02	3.86
33	Myo-Inositol, 6TMS derivative	49.845	2132	2129	0.09	0.13	1.3
34	Phtalic acid derivative*	50.403	2150	-	0.7	0.24	0.03
35	Caffeic acid, 3TMS derivative	50.565	2157	2155	0.89	0.83	0.03
36	Linoleic acid, TMS	51.922	2203	2212	1.31	2.48	4.6
37	13-Octadecenoic acid, (E)-, TMS derivative	52.392	2222	2228	0.17	1.77	0.06
38	3,7,11,15-Tetramethyl-2,6,10,14-hexadecatetraene-1-ol trimethylsilyl ether	52.703	2236	2234	1.01	0.07	0.07
39	2’,6’-Dihydroxy 4’-methoxydihydrochalcone, trimethylsilyl ether	52.717	2418	2405	-	1.99	0.1
40	Pterostilbene, trimethylsilyl ether	57.520	2462	-	0.43	0.77	2.58
41	Pentacosane	58.563	2501	2506	5.85	0.44	0.87
42	Ethyl trans-caffeate, bis(tert-butyldimethylsilyl) ether	58.560	2527	2547	-	3.55	2.22
43	Bisphenol*	59.088	2551		1.65	0.13	0.12
44	1-Docosanol, TMS derivative	59.223	2558	2557	0.19	4.51	4.76
45	Butanoic acid, 4-methoxy-2-nitro-, 2,6-bis(1,1-dimethylethyl)-4-methoxyphenyl ester	59.998	2604	2595	0.72	0.32	0.67
46	Unknown	60.475	2635		0.99	0.84	3.44
47	Behenic acid, TMS derivative	60.550	2645	2644	0.31	0.94	0.83
48	Unknown	60.947	2664		0.95	1.73	2.77
49	Unknown	61.285	2685		3.22	0.69	1.67
50	Maltose, 8TMS derivative, isomer 2	61.422	2693	2693	1.83	3.42	5.26
51	n-Heptacosane	61.537	2700	2700	0.21	0.11	0.31
52	Sucrose, 8TMS derivative	61.705	2712	2712	5.88	0.52	2.61
53	D-(+)-Turanose, octakis(trimethylsilyl) ether, methyloxime (isomer 1)	61.937	2727	2724	0.71	2.66	4.98
54	Maltose, OTMS	62.040	2735	2733	0.29	0.13	0.12
55	D-Cellobiose, (isomer 2), 8TMS derivative	62.262	2749	2762	1.51	0.32	1.3
56	Naringenin, O,O’-bis(trimethylsilyl)-	62.617	2772	2778	1.14	4.1	5.95
57	Isosakuranetin, TMS derivative	63.352	2821	2818	0.94	1.37	2.13
58	Lignoceric acid, TMS derivative	63.650	2842	2838	1.22	1.68	0.44
59	Sakuranetin, TMS derivative	64.215	2882	2877	1.75	0.64	0.11
60	Catechine, 5TMS derivative	64.880	2932	2938	0.33	0.11	0.07
61	Gettibiose, TMS derivative	65.720	2991	2989	0.76	0.36	0.66
62	Triacontane	65.948	3009	3003	0.78	0.4	0.35
63	Hexacosanoic acid, TMS derivative	66.375	3041	3039	0.5	0.69	2.04
64	Unknown	66.975	3085	-	0.66	0.61	0.74
65	Hentriacontane	67.137	3100	3103	0.49	0.23	0.77

*RI* (retention time); *RI_exp_*. and *RI_lit_*. indicate retention indices based on experiments and literature, respectively.

**Table A4:** Percentage of fatty acids in hexane extracts of propolis (HEP) from different regions of Poland: *HEP_P1*—Podlasie (Hajnowka), *HEP_P2*—Masovia (Ciechanow), *HEP_P3*—West Pomerania Province (Miedzyzdroje).

	Substances	RT	RI _exp_	RI _lit_	HEP_P1[%]	HEP_P2[%]	HEP_P3[%]
1	Benzoic acid, methyl ester	15.055	1623	1612	0.69	2.73	0.92
2	Lauric acid, methyl ester	18.820	1877	1804	1.85	2.32	1.28
3	cis-9-Tetradecenoic acid, methyl ester	25.410	2116	2026	5.16	1.94	2.30
4	Pentadecanoic acid, methyl ester	27.530	2194	2108	1.75	0.79	0.70
5	Hexadecanoic acid, methyl ester	29.590	2271	2208	31.17	27.63	20.54
6	Heptadecanoic acid, methyl ester	32.790	2666	2309	9.68	7.67	6.34
7	Octadecanoic acid, methyl ester	34.345	2394	2418	6.40	5.28	4.23
8	Oleic acid, methyl ester	35.050	2488	2434	18.18	25.30	25.90
9	cis-11-Octadecenoic acid, methyl ester	35.565	2512	2468	1.19	0.48	0.81
10	Linolenic acid, methyl ester	37.775	2625	2571	0.95	2.38	5.86
11	Eicosanoic acid, methyl ester	38.180	2651	2639	1.02	1.86	1.89
12	Docosanoic acid, methyl ester	40.790	2844	2835	4.70	5.71	5.00
13	Me. C20:4n3; Eicosa-(8,11,14,17)-tetraenoate <methyl>	41.060	2866	2865	2.48	2.37	2.75
14	Tetracosanoic acid, methyl ester	42.805	3067	3039	14.79	13.91	21.49

*RI* (retention time); *RI_exp_*. and *RI_lit_*. indicate retention indices based on experiments and literature, respectively.
